# Thomas J Henry, as viewed by his son, daughter, and wife

**DOI:** 10.3897/zookeys.796.21056

**Published:** 2017-11-15

**Authors:** Thomas A. Henry, Angela Henry Townsend, Kathryn Henderson

**Affiliations:** 1 Orlando, FL, USA Unaffiliated Orlando United States of America; 2 Kokomo, IN, USA Unaffilaited Kokomo United States of America; 3 Silver Spring, MD, USA Unaffiliated Silver Spring United States of America

When Al Wheeler told me that he was organizing a Festschrift to honor Tom, I (K.H.) thought, how is it possible that it could be time for a Festschrift already? It seems like I just met Tom yesterday. When Al asked me to write an introductory article for the journal, I wondered if I could manage that and called Tom’s children, Tommy (T.A.H.) and Angela (A.H.T.), asking for their help. They both agreed and we began a wonderful collaboration to honor their Dad. As with any life, certain stories and remembrances stand out, and that person’s characteristics and traits are known to some people, but perhaps not to others. We thought about how he enriched our lives through his generosity and guidance and how he influenced our professional lives with sound advice and counsel. We thought about his childhood and the special bond he had with his family, one steeped in the traditions of hard work and a drive to achieve desired goals. We (T.A.H. and A.H.T.) have chosen to focus on some of the highlights of our Dad’s remarkable career and events that show his love of natural history. Included are certain early events, as recounted by our Grandmother.

**Figure 1.**
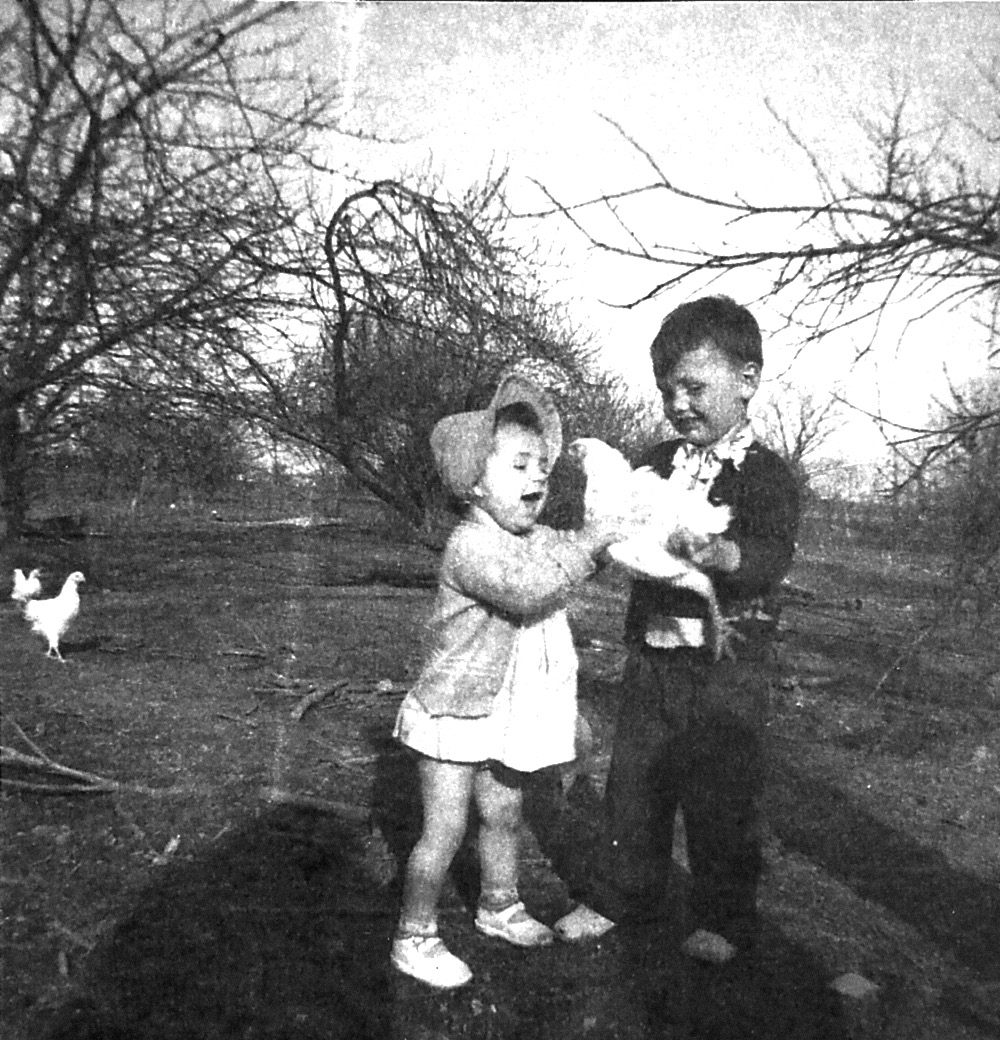
"Here you are the real hero. You are brave enough to hold a real chicken for Debbie to see." (Aunt Phyllis Henry, 1951).

Tom Henry quietly slipped out the back door of his uncle’s farmhouse in rural Indiana. His goal was to catch one of the chickens roaming in the barnyard. Carefully holding the prize chicken in his arms, he proudly showed it to his younger sister, Debbie (Fig. 1). Dad was three years old.

In class at his elementary school, Dad’s name was called over the loudspeaker; he was told to report to the principal’s office immediately. From there, he was sent home. As our Grandmother told the story, every surface in the house was moving—the drapes, the walls, even the upholstery. The praying mantis egg cases that he had collected and kept in his bedroom had all hatched. He was the most popular kid at school the next day when he brought in newly hatched praying mantids for the teacher to raise in the classroom. Dad was ten years old.

Other childhood stories could be told, but one common factor is consistent throughout them all: our Dad loves nature. From his earliest years to the present, he has pursued his interest in nature through his studies, his work, and his hobbies. His love of the natural world quickly developed into his true passion, the study of insects.

Thomas Joseph Henry was born in Logansport, Indiana, a small town between the Wabash and Eel Rivers. His parents, Joseph Fouts Henry and Betty Vitello Henry, both grew up in the area and remained there to raise their family. Dad is the eldest child, and most of his siblings and extended family still reside in the area.

Growing up in a small town allowed Dad tremendous freedom. Both Logansport and the Henry family farm provided perfect places for a kid to explore and pursue great adventures and to collect specimens for the insect collection he began as a child. Dad still has a small, spiral-bound notebook he used for field notes that included dates of collection, brief descriptions, and measurements in millimeters. His interest in natural history, however, was broad. He was equally fascinated with skeletons of animals he found, as well as live animals and plants.

Dad would spend long summer days wading or floating on his raft down the Wabash River fishing, observing plants and animals, and always looking for and collecting insects. The emergence of praying mantids aside, our Grandmother was a huge influence in Dad’s life. Although she may not have always understood his interests in natural history, throughout his childhood she always encouraged and supported his efforts. Fish tanks, snakes, turtles, and insects were all welcomed as long as nothing else escaped from his bedroom to other parts of the house. As he grew older, she continued to support him and was extremely proud of his many accomplishments.

By his early high school days, Dad was well regarded for his achievements in academics and sports (with five varsity letters). A teacher familiar with Dad’s knowledge of biology and insects suggested that he apply to Purdue University to study entomology. At the time, Dad did not know that the study of entomology could eventually lead to a paid position. The encouragement from that teacher propelled Dad on his career path, one he’s never wavered from.

In 1972, we were two- (A.H.T.) and four-years-old (T.A.H.) when our family moved to Pennsylvania. Dad had been hired as an insect taxonomist with the Pennsylvania Department of Agriculture (PDA) in Harrisburg. While still working in Harrisburg, he completed a master’s degree at The Pennsylvania State University. Two or three times each week during the fall and spring semesters, he made the 180-mile round trip to attend classes.

Dad met Al Wheeler (Dr. Alfred G. Wheeler) at the PDA, and they have remained good friends and colleagues to this day. Throughout the years, they have collaborated on numerous projects. It is with great appreciation and affection that we thank Al for his support and hard work in making this journal issue possible.

In 1980, Dad accepted a position as Research Entomologist with the U.S. Department of Agriculture, Systematic Entomology Laboratory, at the Smithsonian’s National Museum of Natural History in Washington, DC. He earned his Ph.D. from the University of Maryland while working in the SEL. He remains there today, still as focused on Heteroptera as he was when he began there thirty-seven years ago. Early in Dad’s career with the USDA, he became close friends with Dick Froeschner (Dr. Richard C. Froeschner), a Smithsonian colleague. Together they edited the *Catalog of the Heteroptera, or True Bugs, of Canada and the Continental United States*, a significant work for both of them that was published in 1988.

He met his wife, Katy, on a blind date over thirty years ago, and that meeting began a whole new chapter in both their lives. We met her a few weeks later and knew it was a serious relationship even then, possibly before they did. They were certainly made for each other. Today they are still partners in everything and inspiring for us to watch.

Although our interests led us to professions different from our Dad’s, we have the same drive and passion to succeed as he does. He was lucky enough to know as a young child what he loved to do and was able to turn that love into a life-long career.

Over the years, we have had to explain to some people what the word entomology means and what our father does in his work. Responses have varied. Some people are fascinated by his work, but others wonder how anyone could work with insects. We lovingly refer to him as a “Rock Star” of entomology, a title one of us (T.A.H.) bestowed on him years ago. All three of us are very proud of him and are honored that he named a new insect species after each of us: *Epipolops angelae*, *Epipolops thomasi*, and *Epipolops kathrynae*.

Dad’s contributions, research, and publications are well documented. What isn’t as well known is his patience and kindness to others, including colleagues and professional staff, interns and volunteers, and friends and family. He is always willing to help, whether it involves a work-related task or assisting a neighbor in need. Also, he is an excellent listener. Months after a conversation, he might inquire about something that was said, wanting to know if an issue had been resolved and if the outcome was good.

**Figure 2.**
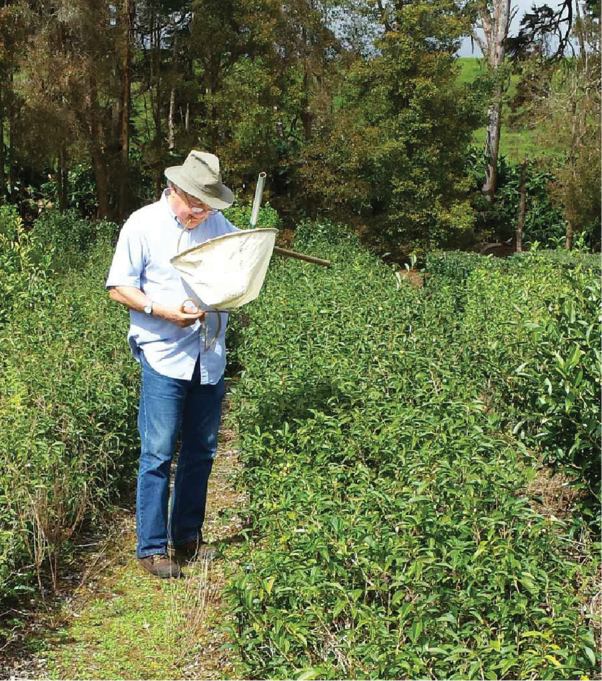
Tom Henry collecting Heteroptera in Hawaii, 2017.

**Figure 3.**
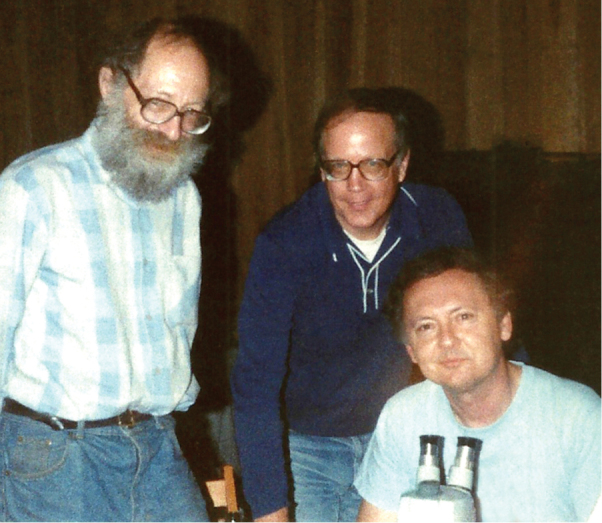
Left to right: Izya Kerzhner, Al Wheeler, and Tom Henry working in the West Virginia cabin, 1994.

We know that nothing at all will change for Dad if he ever decides to retire. He will continue his research and writing just as he has always done. He will continue to travel the world to collect and identify new species of insects (Fig. 2). He will attend conferences and serve on panels and give presentations just as he has throughout his career. He will continue to acquire new colleagues, specifically heteropterists with whom he will willingly share his knowledge of the group. Other interests will also continue to be pursued. He loves to work in his beautiful yard and gardens; each spring he plants a large vegetable garden that produces an abundance of his favorite onions and hot peppers. For years he has maintained freshwater aquariums (twenty-six and counting), where he breeds tropical fish and grows aquatic plants. He will continue building and completing projects on his cabin, a quiet retreat in the mountains of West Virginia (Fig. 3). Dad has always found more time in a day than most other people do. His enthusiasm for life and dedication to his work are, in our eyes, unsurpassed.

But, given all of his interests, one fact is indisputable. If you need to find our father, Dr. Thomas J. Henry, either now or eventually in retirement, chances are that he will be following his truest passion, the study of Heteroptera, in a world he was born to be in, doing what he loves best for as long as possible.

